# Cost Effective Mobile Mapping System for Color Point Cloud Reconstruction

**DOI:** 10.3390/s20226536

**Published:** 2020-11-16

**Authors:** Cheng-Wei Peng, Chen-Chien Hsu, Wei-Yen Wang

**Affiliations:** Department of Electrical Engineering, National Taiwan Normal University, Taipei 106, Taiwan; 80875003h@ntnu.edu.tw (C.-W.P.); wywang@ntnu.edu.tw (W.-Y.W.)

**Keywords:** HD map, color point cloud, mobile mapping system (MMS), autonomous driving

## Abstract

Survey-grade Lidar brands have commercialized Lidar-based mobile mapping systems (MMSs) for several years now. With this high-end equipment, the high-level accuracy quality of point clouds can be ensured, but unfortunately, their high cost has prevented practical implementation in autonomous driving from being affordable. As an attempt to solve this problem, we present a cost-effective MMS to generate an accurate 3D color point cloud for autonomous vehicles. Among the major processes for color point cloud reconstruction, we first synchronize the timestamps of each sensor. The calibration process between camera and Lidar is developed to obtain the translation and rotation matrices, based on which color attributes can be composed into the corresponding Lidar points. We also employ control points to adjust the point cloud for fine tuning the absolute position. To overcome the limitation of Global Navigation Satellite System/Inertial Measurement Unit (GNSS/IMU) positioning system, we utilize Normal Distribution Transform (NDT) localization to refine the trajectory to solve the multi-scan dispersion issue. Experimental results show that the color point cloud reconstructed by the proposed MMS has a position error in centimeter-level accuracy, meeting the requirement of high definition (HD) maps for autonomous driving usage.

## 1. Introduction

**Figure 1:**
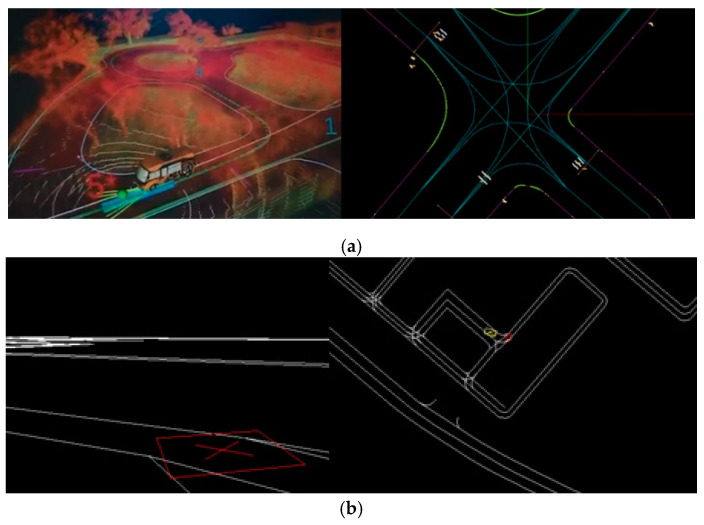
(**a**) Autoware HD map: (Left) 3D view; (Right) 2D view; (**b**) nVIDIA Driveworks HD map: (Left) 3D view; (Right) 2D view.

High definition maps (HD maps) play a crucial role in the development of autonomous driving systems. Thus, many well-known autonomous driving platforms, including Autoware [1] and nVIDIA Driveworks [2], have been actively defined and developed HD maps, as shown in Figure 1. A HD map consists of a point cloud and road/lane vectors with semantic information. With a point cloud, a Lidar sensor plays an important role to assist localization algorithms to enhance the positioning performance achievable with GNSS/IMU receivers. It is therefore important to have an accurate point cloud working together with GNSS/IMU to ensure reliable self-localization ability for autonomous driving.

In order to deliver end-to-end transportation services, essential road/lane vectors with semantic information in the HD map are indispensable. More specifically, autonomous vehicles trace and drive along the lane center lines which consist of geodetic location points. Road/lane boundaries are also provided to help runtime calculation for obstacle avoidance in the sight and make detour or stop decisions afterward. This kind of process is called local planning. Different from local planning, global planning aims to achieve a route mission between one point to another. It occurs before a vehicle starts to move, and sometimes happens when a detour case occurs. The process of the global planning focuses on the nodes at the two end points of each road vectors. Each node contains pre-stored traffic rules, for example: one-way, turn restriction, car type allowance, etc. By applying routing algorithms (A-star for example), suitable and available nodes between a start and destination point can be determined. In summary, an accurate color point cloud not only helps an autonomous vehicle to localize itself, but also provides an important basis to produce road/lane vectors with semantic information in HD maps for local/global route planning.

Thanks to their advantages of mobility to survey a large 3D area, MMSs have gained great popularity for color point cloud reconstruction over the past years. Well-known brands like Rigel [3], Leica [4], Optech [5], Trimble [6] have had MMS products on the market for several years. These MMSs are generally equipped with high performance computing platforms for data preprocessing, high-grade GNSS/IMU for positioning, survey-grade Lidar for ranging, and high-resolution camera for color registration.

**Figure 2:**
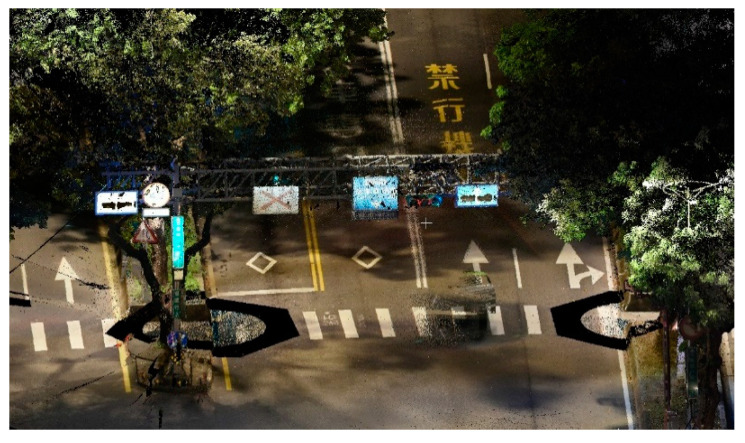
Color point cloud reconstruction by a RIEGL 3D Terrestrial Laser Scanner [3].

With the use of high-end equipment, a rigid body frame setup, and brand-own post-processing software, high-accuracy color point clouds can be generated for use in a variety of applications, including road inventory management, heritage conservation surveys, etc. Figure 2 shows a color point cloud reconstructed by a Rigel 3D Terrestrial Laser Scanner [3]. Unfortunately, high-end equipment and proprietary software inevitably involve a high in use cost, greatly preventing practical autonomous driving implementations from being affordable.

**Figure 3:**
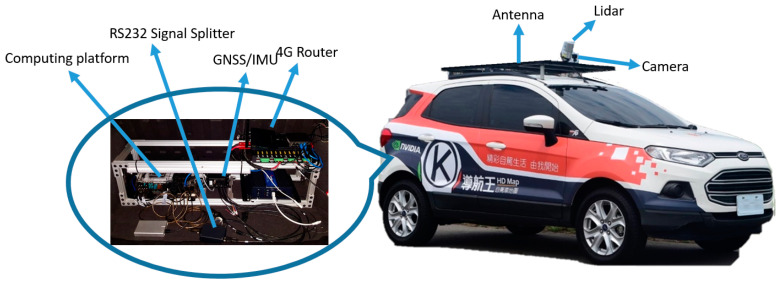
Key components of the proposed MMS.

As an attempt to solve the abovementioned problems, in this paper, we present a low-cost and high-efficiency MMS to generate an accurate color point cloud as shown in Figure 3, in which a GNSS/IMU, a consumer-grade Gigabit Multimedia Serial Link (GMSL) camera, and a non-survey grade Lidar were only used, thus significantly reducing the cost. To enhance GNSS/IMU positioning, the Virtual Base Station Real Time Kinematic (VBS-RTK) correction data is offered via a 4G connection in our integrated MMS platform. Through GNSS/IMU and Lidar extrinsic calibration, Lidar Direct Georeferencing (DG) ability of the proposed MMS is achieved. By obtaining the rotation and translation matrices between the Lidar and camera through a calibration process, pixels in the image can be matched with Lidar points. Thus, we can register RGB attributes to the corresponding Lidar points with global coordinates in the end. This method is much straightforward and time efficient without surface extraction in the offline process [7]. Additionally, to improve the geo-location accuracy of the color point cloud, we employed a number of control points to fine tune the point cloud. According to the experimental results, the color point cloud reconstructed by the proposed MMS has a position error in centimeter-level accuracy, meeting the requirement of HD map for autonomous driving usage.

This paper is organized as follows. Section 2 discusses related works on color point cloud reconstruction. Section 3 describes the hardware configuration of the proposed MMS. Section 4 presents the proposed approach for a cost-effective color point cloud reconstruction, where key function blocks are described in more details. Section 5 shows the experimental results that we conducted using the proposed hardware and methods. Finally, we conclude this paper in Section 6.

## 2. Related Work

Lidar point clouds have been widely discussed for extracting road features [8,9] in various manners. Jeong [8] presented an intensity correction flow to enhance the feature intensity due to the distance from the moving platform and the angle of incidence. Niijima [9] presented a method combining 3D point clouds with fundamental geospatial data information, because aligning road features with a graph-based map could add stability to overcome the errors caused by the feature extraction process. Nagy [10] proposed a voxel-based convolutional neural network (CNN) architecture which has the ability to remove the impact of the phantom phenomenon for point clouds collected on the open roads. Zhao [11] combined a 3D Lidar and camera and utilized a CNN model to detect objects for autonomous driving usage. The segmentation phrase in [10,11] is an essential process to make their methods work. Hence, integrating both color and intensity information into the point cloud is believed to enhance the segmentation result in related methods. On the other hand, conventional Lidar point clouds are widely utilized for vehicle localization purposes. Wan [12] presented a multi-sensor fusion architecture which integrates GNSS, IMU and Lidar to estimate the best position result via an error-state Kalman filter for fusing measurements. Shamseldin [13] proposed a Pseudo-GNSS/INS framework for collaborating with Lidar simultaneous localization and mapping (SLAM) to determine positioning in GNSS-denied areas. In [12,13], the localization performance highly relied on the accuracy of Lidar point cloud. From the above discussions, we conclude that the trend for future research is the use of color point clouds and accurate point clouds are able to assist with localization.

Basically, color point cloud reconstruction can be categorized into static-based and mobile-based approaches. For static-based color point cloud reconstruction, Neubauer [14] used a terrestrial laser scanner with a calibrated digital camera for high accuracy, high resolution and long distance topographic scanning in archaeology. Moussa [15] presented an automatic procedure by combining digital images and laser scanner data to have a full representation of a scene by generating highly virtual reality models. Compared with [14,15], An [16] presented a cost effective solution of color point cloud system by integrating a 2D Lidar and camera with their proposed calibration process. However, this approach exhibits some limitations if used in an outdoor environment. Zhang [17] presented a statistical method to filter out noises in color point clouds by using voxel boundaries. This approach is able to enhance the color correctness in [16] with a minor color registration error due to the calibration process. Jo [18] combined terrestrial laser scanning and unmanned aerial vehicle (UAV) photogrammetry to establish a three-dimensional model of a heritage temple, where the UAV provided different view angles to compensate the terrestrial scan to avoid blind angle in large-object construction.

As for mobile-based color point cloud reconstruction researches, Hulková [19], Yastikli [20] and Alsadik [21] have presented their mobile-based laser scanning systems in the heritage and highway documentation fields, respectively. Amano [22] presented a full color 3D point cloud construction from a quadcopter, which combines Lidar scans and camera images for use in disaster and accident investigation. In a vehicle-borne mobile based land survey, Zeng [23] proposed a mobile measurement system employing a Lidar and a panoramic camera with rigid sensors setup to assign color attributes into Lidar scan. Yao [24] proposed a mobile mapping system by combining multiple 2D Lidars and a panoramic camera for registration, by using feature points to limit the point cloud coincidence error. Vechersky [7] proposed a different method to solve the problem of color registration errors, by taking non-rigid loose coupling of sensors to solve a number of challenges related to timing, point visibility and color determination problems. From the experimental results, this method can solve most of the problems particularly when dealing with the non-rigid camera and Lidar setup. However, there exist heading and time alignment problems, causing color mis-registration as mentioned in the paper. Based on the above-mentioned works, it can be seen that MMS is the mainstream platform to establish color point clouds. To achieve high-accuracy Lidar Direct Georeferencing ability, most MMSs integrate GNSS and IMU [25,26,27,28,29] to improve the positioning capability. To further enhance the accuracy, many MMSs integrate additional sensors like wheel encoders or e-compasses to provide extra observation data to constrain the position errors. As a result, the positioning system is able to maintain a certain level of accuracy under critical signal condition via various filtering mechanisms based on observation and prediction workflow. These approaches, unfortunately, could not provide accurate positioning results by GNSS/IMU for cases where satellite signals are weak. Hence, the SLAM technique is believed to enhance the positioning ability. Visual SLAM is a major stream in the research field, including stereo vision approaches [30], stereo with wide baseline approaches [31] as well as localization precision improvement in GNSS-denied areas [32]. Different from visual SLAM, Lidar SLAM [33,34,35] is implemented to compensate and gain better position reliability in open roads, where the features in urban environments can be sufficiently extracted. From the abovementioned discussions, it can be found that performance of available MMSs has been improved in various aspects over the past years, but there is still room for investigation to construct an accurate color point clouds by GNSS/IMU with commercial sensors, in which plenty of hardware and software integration and optimization are required, including derivation of extrinsic parameters between sensors, registration of color attributes into point cloud, and trajectory refinement, etc. We will provide feasible solutions to address the above issues in the following sections of this paper.

## 3. MMS Hardware Architecture

**Figure 4:**
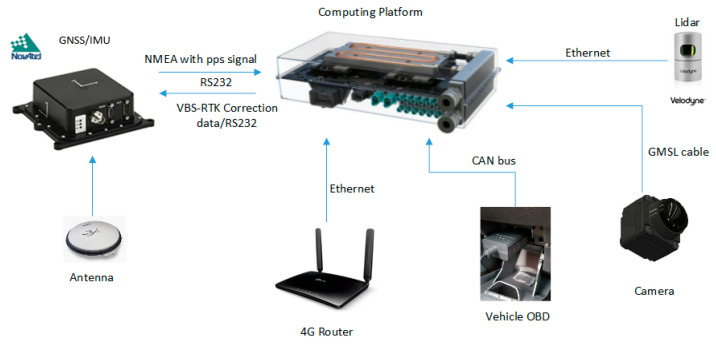
Hardware architecture of the proposed MMS.

A typical MMS generally comprises devices that collect geospatial data from a vehicle platform, typically fitted with a position system, computing platform, and remote sensors [26,28]. The MMS hardware architecture proposed in this paper for data collection is shown in Figure 4. Except for the sport utility vehicle (SUV) used as the vehicle platform, the remaining components are described in details as follows:

### 3.1. Positioning System

A SPAN-IGM-A1 GNSS/INS positioning system (NovAtel, Calgary, AB, Canada) is employed to obtain the position as an initial reference for the Lidar sensor. The position module comprises a tightly coupled RTK GNSS/INS engine. Through a 4G network connection, the virtual based system-real time kinemics (VBS-RTK) correction data can significantly reduce the position error and height error to 2 cm and 5 cm (90% circular error probability), respectively, according to our real tests under open sky conditions.

**Figure 5:**
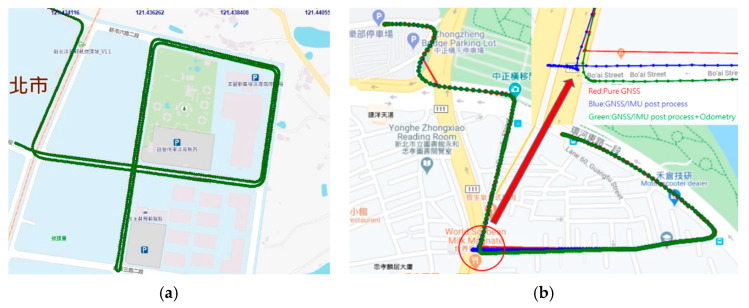
(**a**) Position trajectories in open area; (**b**) Position trajectories in urban area. Red: Pure GNSS position is unreliable, Blue: A post process integrating the IMU is used to reduce the position error, Green: Speed information is considered in the post-process to further reduce the position error as indicated in the red circle.

**Figure 6:**
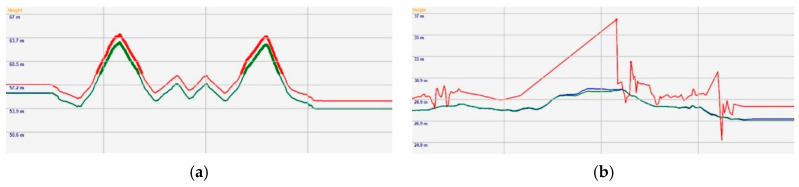
(**a**) Altitude result in open area; (**b**) Altitude result in urban area. Red: Pure GNSS, Blue: GNSS with IMU and VBS-RTK after post processing, Green: GNSS with IMU, VBS-RTK and Car speed information after post-processing.

In rural areas, GNSS signals works well with the VBS-RTK correction data. Thus, the 3D position error of GNSS is small and the trajectory is smooth, which is desirable for Lidar point cloud reconstruction. In urban or suburban environments, however, the GNSS satellite signals can be deteriorated by multipath effects due to signal reflection, resulting in inaccurate observation data. Therefore, even though SNR of the satellite signal strength is good, multipath effects could result in position errors that VBS-RTK correction data cannot improve. Given the fact that GNSS system with VBS-RTK might not be reliable in urban areas, an inertial measurement unit (IMU) and car speed information can be used to reduce the position error, keeping the trajectory as smooth as possible. In this paper, we utilized a licensed post-processing software to generate location points of the trajectory at 200 Hz and converted the coordinate format to 2-degree transverse Mercator (TM2) based on a Taiwan Datum 1997 (TWD97) system for further usage. Figure 5 shows the position trajectories in open and urban areas, respectively, where the integration of IMU and speed information by a post processing step is able to reduce the position error and make the trajectories much smoother. On the other hand, Figure 6 shows the comparison of altitude results of GNSS with and without VBS-RTK and car speed information. As demonstrated in Figure 5 and Figure 6, GNSS alone provides inaccurate positions, particularly in urban areas. To reproduce a smoother trajectory, a post-processing step taking into account the IMU and vehicle speed information would be preferred for the point cloud reconstruction (green line in Figure 5). One thing we should keep in mind is that IMU and speed information accumulate angular and velocity errors as time elapses. That means an accurate position should be resumed before the accumulated position error becomes unacceptable. Otherwise, a higher-grade IMU would be needed for employment to reduce the errors. However, it would inevitably bear an extremely high cost. Thus, we prefer to use a Lidar-based NDT SLAM to refine the trajectory to achieve the objective to establish a low-cost MMS in this paper.

### 3.2. Sensors

In this paper, a Velodyne-32E [36] 3D Lidar with a range accuracy of less than 2 cm is used for the measurement task to collect point cloud data. The sensor itself consists of 32 laser emitters which are vertically fired in a sequential manner. With a spinning mechanical design, the sensor has a 360-degree horizontal field of view (FOV) with configurable revolutions per minute. The measurement data can be logged via a specified port by UDP connection. In our system, the Lidar timestamps by default were synchronized with GPS every second via the pulse per second (PPS) signal. The firing angle and spin angle are well controlled so that we are able to obtain the precise time of each Lidar point, minimizing the spatial transformation error when the MMS is moving. In other words, we are able to align each Lidar points with GNSS trajectory by timestamps to convert each individual Lidar points from sensor coordinates to geo-coordinates.

As for the camera sensor, we employed a sf3324-10x Gigabit Multimedia Serial Link (GMSL) camera (Sekonix, Dongducheon-si, Gyeonggi-do, Korea) with 120° FOV and 30 fps in the proposed MMS. A GMSL serializer, as the name implies, of the camera can provide gigabits per second bandwidth to transmit raw data from the image sensor by working together with a deserializer on the computing platform. The communication between the serializer and deserializer supports the high-bandwidth transmission and data integrity requirements to achieve dual direction communication and low latency of data transmission time in the system. Additionally, the trigger mode is enabled in this camera sensor. Thus, timestamp of each video frames can be ascertained when the image frames are captured. In comparison to conventional USB cameras, a GMSL camera is much more suitable to achieve time-sensitive integration in the proposed MMS.

### 3.3. Computing Platform

A Drive PX2 (nVIDIA, Santa Clara, CA, USA) is employed as the computing platform in this paper, which includes dual SoCs on the platform. The power consumption of the computing platform is 250 W. The benefits of employing the PX2 lies in that it not only saves space in the trunk but also the cost of an extra battery, which is no longer required because the power of the original car setup is sufficient to meet the power consumption needed for the entire system. The PX2 can handle all the sensory data as well as position data logged via the compact computing platform. It is extremely suitable for applications which need powerful computation within a limited physical space like autonomous driving cars. Thanks to the camera driver, video frames can be saved and logged with their timestamp with micro-second resolution on the platform.

In this section, we have described the sensors and major components we employed in the proposed MMS platform. A post-processing step considering GNSS/IMU trajectory with speed information is used to solve the problem of GNSS/IMU positioning when satellite signals are weak or unavailable. The Lidar and camera we employed in this paper are controlled with accurate time synchronization. Hence we can expect this hardware configuration to be capable of achieving the objectives of low-cost and high-efficiency realization. To leverage the loading of the computing platform, Lidar, speed data, IMU and GNSS data are stored and handled by one of the SoCs on the platform called Tegra A, while the other SoC called Tegra B is in charge of communicating with the deserializer to fetch the video frames and their corresponding timestamps. To this end, we are able to collect sufficient data for further processing by a post process to reconstruct a color point cloud.

## 4. Color Point Cloud Reconstruction

**Figure 7:**
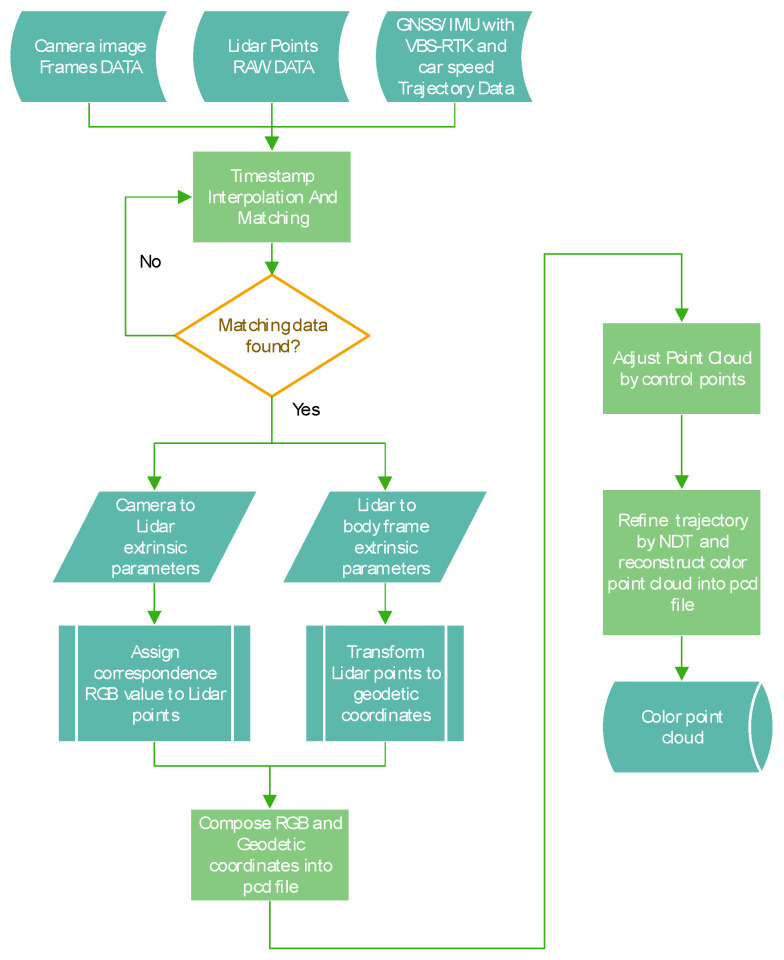
Flowchart of the proposed color point cloud reconstruction.

Figure 7 shows the flowchart of the proposed approach for a cost-effective color point cloud reconstruction, where the main processes comprise: Timestamp interpolation and matching, Compose RGB and geodetic coordinates into pcd file, Adjust point cloud by control points, Refine trajectory by NDT and reconstruct color point cloud into a pcd file.

### 4.1. Timestamp Interpolation and Matching

**Figure 8:**
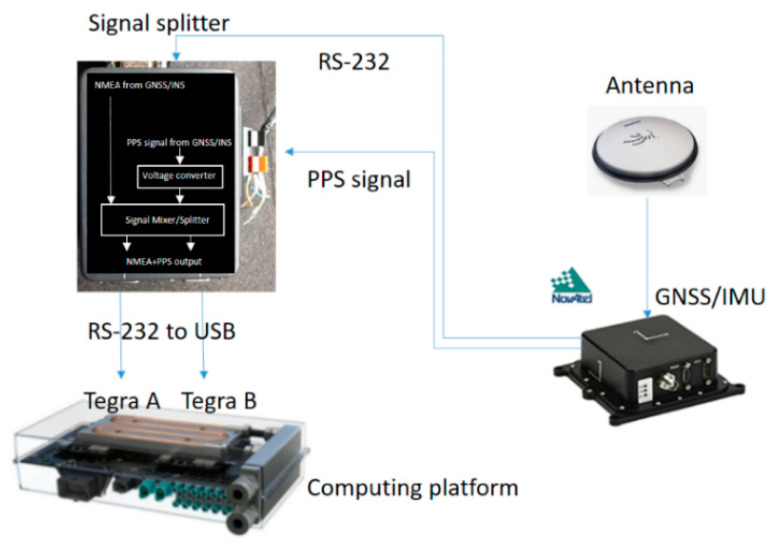
Schematic diagram showing PPS time correction with a signal splitter box.

In the proposed MMS, Lidar’s timestamps have been synchronized with GNSS. Hence, camera frames and vehicle speed data have to synchronize with the Lidar’s timestamps by the same method. It is therefore critically important to obtain time drift of the computing platform before synchronization can be performed. The GNSS time can be derived by parsing National Marine Electronics Association (NMEA) sentences. However, the time contained in the NMEA sentence only represents the time when satellite signals are decoded by GNSS receiver. The slight time difference between the computing platform and the GNSS receiver should be considered to make accurate time correction. In order to solve the slight and varying delay time, a PPS signal as shown in Figure 8 is essential for accurate time correction for sensors in MMSs [26]. The PPS signal is generated independently from the receiver when the timestamp frame from satellite signals is decoded. Hence, we integrate the PPS with NMEA physical RS-232 port into a signal splitter box and connect to the computing platform through RS-232 to USB cable. On the computing platform, we develop an interrupt utility to detect the PPS signal every second to obtain the system time of the computing platform, which is then deducted by the PPS time to finally obtain the time difference for accurate correction.

**Figure 9:**
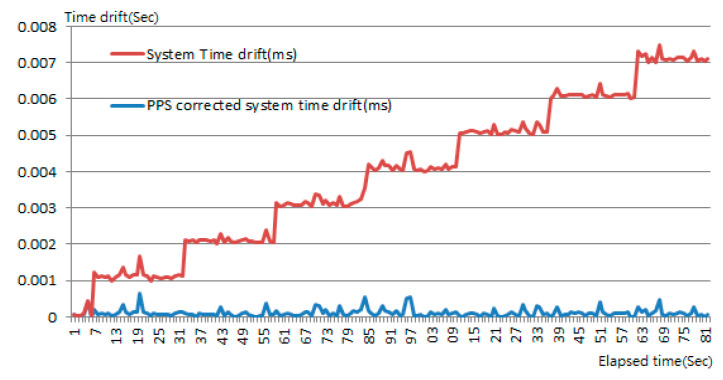
Correction to system time drift using the external PPS signal.

According to the experiments, the computing platform has 1 ms drift in about 25 s as time elapses. Thus, the approach is able to correct the system time to an accuracy within 1 ms. Figure 9 shows the correction to system time drift using the external PPS signal indicated by a blue line, while the time drift without correction is indicated by a red line. Thanks to the precise synchronization, we are able to simply take the Lidar point timestamp to match with the nearest camera frame. Once a desired correspondence between Lidar point and camera frame is found, an accurate interpolation point between two GNSS positions can be found by the Lidar point timestamp. Otherwise, we skip the current Lidar point and then proceed to next Lidar point for matching.

### 4.2. Compose RGB and Geodetic Coordinates Into Pcd File

**Figure 10:**
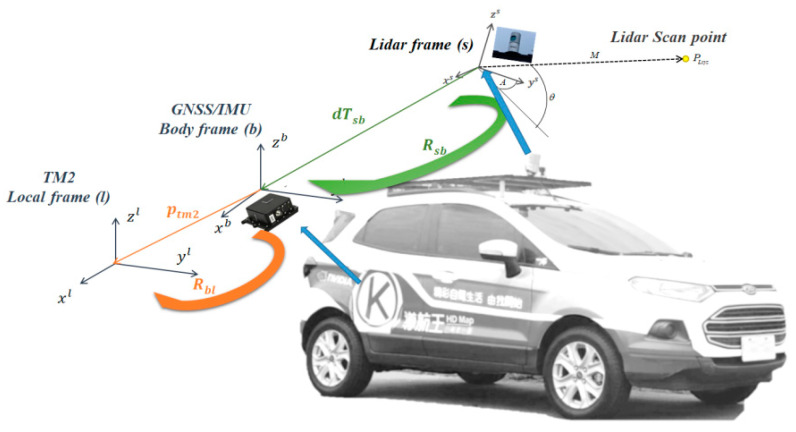
Schematic diagram showing transformations from Lidar frame to local frame.

According to Lidar sensor specifications, Lidar data packets can be sequentially obtained via Ethernet. By analyzing each data packet, emitting timestamp, azimuth, return distance and intensity of each Lidar channel can be obtained. Figure 10 is a schematic diagram showing transformations from Lidar frame to local frame The coordinate of the points, $P_{Lxyz}=(P_{Lx},P_{Ly},P_{Lz})$, in Lidar frame can be expressed by the following equations:

(1) $$P_{Lx}=M\times \cos\theta \times \sin A$$

(2) $$P_{Ly}=M\times \cos\theta \times \cos A$$

(3) $$P_{Lz}=M\times \sin\theta ,$$

where $P_{Lx}$, $P_{Ly}$ and $P_{Lz}$ are the coordinates of the 3-dimensional Lidar points $P_{Lxyz}$, M is the return distance,θ is the Lidar channel angle, and A is the azimuth angle. In this paper, we omit M if it is greater/equal than 25 m to reduce inaccurate coordinate transformations in the result.

In order to register the geodetic coordinates in the point cloud, the location points along the trajectory are linearly interpolated to align each Lidar channel emitting timestamp in a precise manner. Hence, considering interpolated location points and rotation, translation between IMU and Lidar, the geodetic coordinate of Lidar points can be expressed as:

(4) $$p_{GTM2}=p_{TM2}+R_{bl}(R_{sb}P_{Lxyz}+dT_{sb}),$$

where $p_{GTM2}$: Lidar point in TM2 coordinate format based on TWD97 system; $p_{TM2}$: GNSS/IMU location point in TM2 coordinate format based on TWD97 system; $R_{bl}$: rotation matrix from body frame to local frame (TM2); $R_{sb}$: rotation matrix from Lidar sensor to body frame (IMU); $P_{Lxyz}$: coordinate of the 3-D Lidar points in Lidar frame and $dT_{sb}$: offset between the Lidar sensor and GNSS/IMU center.

In this paper, we assume the extrinsic parameters between the Lidar and GNSS/IMU are known. Hence, the geodetic coordinate of each Lidar point can be determined by Equation (4). Additionally, the intrinsic parameters of the camera are successfully obtained by the OpenCV utility. Next, we find the relationship between the camera and Lidar by applying an open utility for camera and Lidar calibration called Calibration Toolkit [37]. The calculation of rotation matrix between Lidar and camera is shown in Equations (5)–(7):

(5) $$R^{*}\cdot N=M,$$

(6) $$R^{*}\cdot (N\cdot N^{\prime })=M\cdot N^{\prime },$$

(7) $$R^{*}=(M\cdot N^{\prime })\cdot {\left(N\cdot N^{\prime }\right)}^{-1}\;\mathrm{if}\;\mathrm{rank}(N\cdot N^{\prime })=3,$$

where $R^{*}$: rotation matrix between the camera and Lidar; N: matrix formed by all normal vectors on chessboards in camera coordinates and M: matrix formed by all normal vectors on chessboards in Lidar coordinates.

Once the rotation matrix $R^{*}$ is obtained, an optimization formulation to obtain the translation vector $T_{\left(x,y,z\right)}$ is as follows:

(8) $$\underset{\left(x,y,z\right)}{\arg\min}\sum_{i}\sum_{j}({\left(R^{*}\cdot p_{i}+T_{\left(x,y,z\right)}-q_{i,j}\right)}^{\prime }\cdot (R^{*}\cdot n_{i}){)}^{2},$$

where $T_{\left(x,y,z\right)}$: translation matrix; $p_{i}$: position of the *i*-th chessboard in camera coordinates; $n_{i}$: normal vector of the *i*-th chessboard in camera coordinates and $q_{i,j}$: *j*-th point on the *i*-th chessboard in Lidar coordinates.

**Figure 11:**
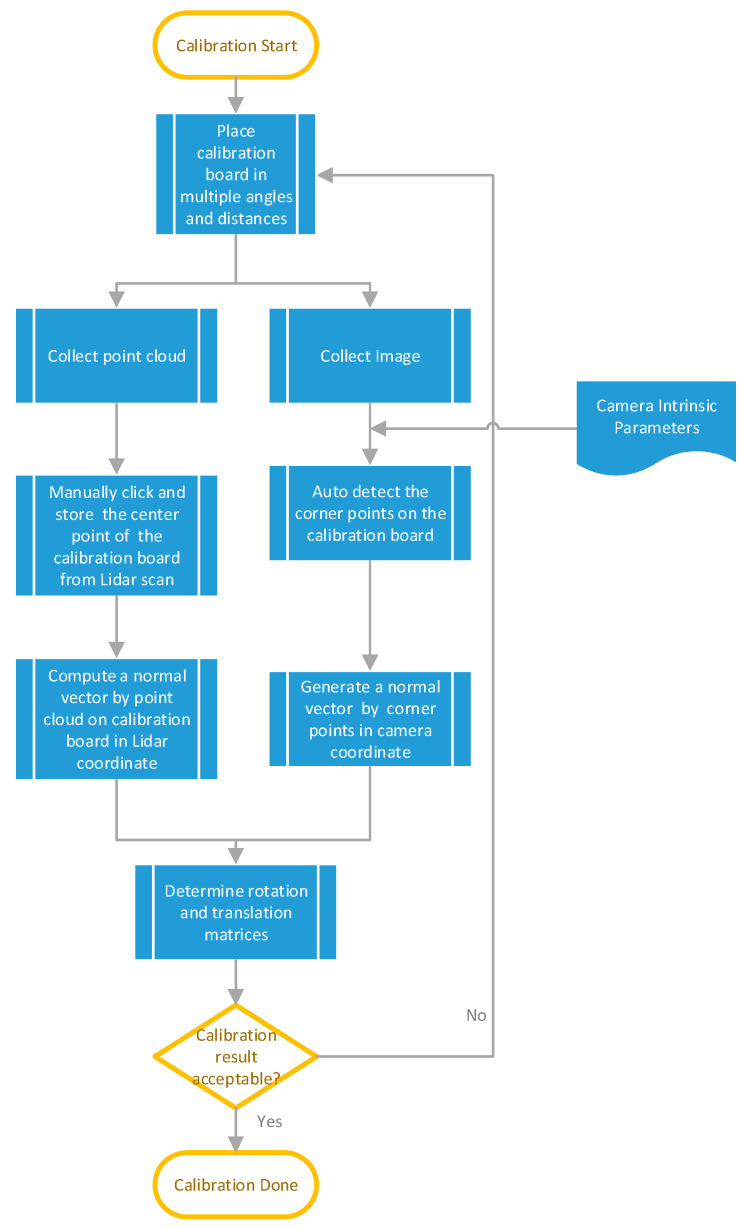
Flowchart of extrinsic calibration between camera and Lidar sensors.

The calibration requires users to manually click the center of the calibration board in the Lidar scan window. The normal vectors of the Lidar scans and camera images on the calibration board can be obtained to calculate the rotation (Equation (7)) and translation (Equation (8)) matrices between camera and Lidar coordinate systems. Figure 11 shows the flowchart of extrinsic calibration between camera and Lidar sensors, where the calibration process starts by placing a calibration board in front of the camera and Lidar sensors in various angles and distances. Through calibration utility, rectangle anchors on the image can be automatically recognized to determine the center position of the calibration board in the camera coordinate system. As for Lidar, we prefer as many points as possible on the calibration board for each frame scan to obtain an accurate plane of the calibration board.

Note that we calibrate the sample data several times with different distances and angles to cover as large field of view as possible to obtain better calibration results, so that the deviation of the normal vectors to the calibration board are statistically converged to obtain more accurate translation and rotation matrices. The calibration result could be unsatisfactory if there is a deviation in clicking the center of the calibration board from the Lidar scan.

**Figure 12:**
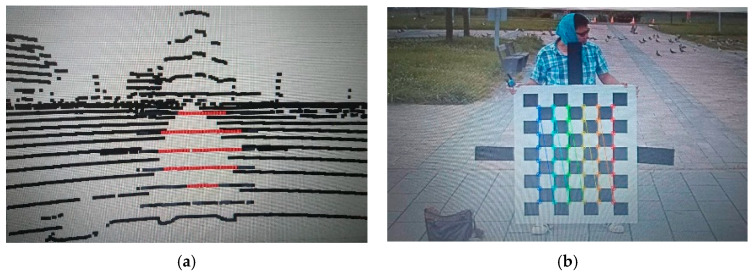
Calibration between Lidar scan and camera image (**a**) Lidar scan on calibration board (**b**) image of calibration board.

**Figure 13:**
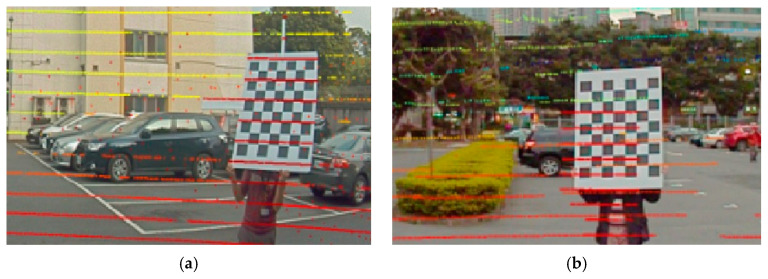
Calibration results: (**a**) desired, (**b**) unacceptable because of inaccurate translation and rotation matrices.

Figure 12 shows the calibration process of a multi-sensor unit consisting of a camera and a Lidar to independently capture images and Lidar scan data at the same time to derive the translation and rotation matrices. Figure 13a shows a desired calibration result, and Figure 13b is unacceptable because of the inaccurate translation and rotation matrices.

In the proposed MMS, the camera captures images at 30 fps and the Lidar has a spin rate of 15 Hz. By searching the nearest camera timestamp from Lidar point timestamps within +/−15 ms, we can match the camera frame with Lidar points in pairs. We then utilize the calibration parameters to compose the RGB attributes to the corresponding Lidar points. Otherwise, we treat the Lidar points not able to match and discard the points. This process outputs the color point cloud in file format ‘point cloud data (pcd)’.

### 4.3. Point Cloud Adjustment by Control Points

GNSS/IMU trajectory error will lead to position errors of each point in the color point cloud. Therefore, it is necessary to have a fine-tuning process to adjust the positions of the color point cloud. In order to adjust the color point cloud, we use a handheld GNSS RTK receiver and total station to establish control points to make adjustment to the point cloud constructed in the survey area. A set of feature points (for example anchor points of arrows or lane dividers) uniformly chosen inside the survey area are recommended.

**Figure 14:**
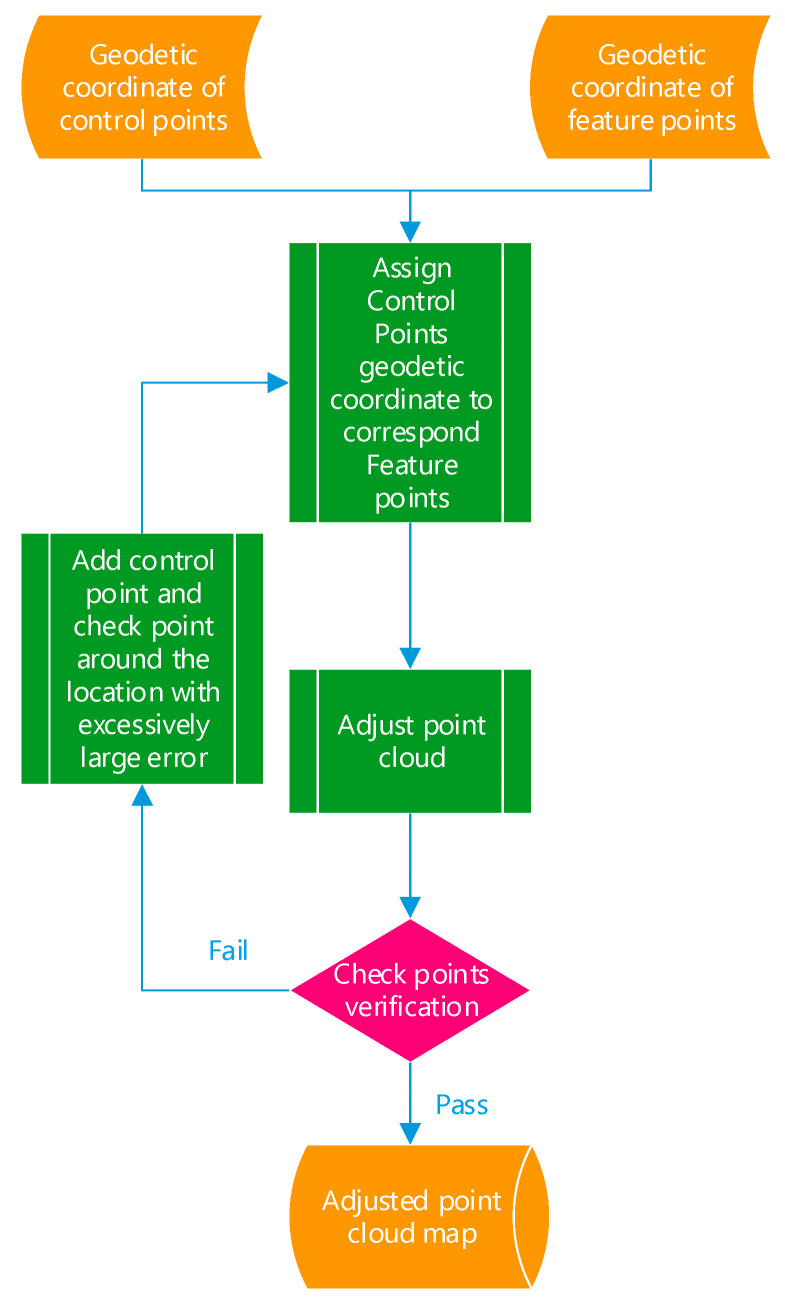
Point cloud adjustment via control points.

After an outdoor survey, we divide the feature points into two point sets, where one is control points and the other is check points. Figure 14 shows the flowchart of point cloud adjustment via control points. A manual labeling is performed by assigning the geodetic coordinate of the control points to their corresponding feature points in the point cloud. The other Lidar points are respectively adjusted according to the distance between the nearest control point and its corresponding feature point. In this paper, a weighting parameter is introduced for position adjustment, where Lidar points with a larger distance from the nearest control point have a lesser effect on position adjustment.

Finally, a set of check points is used to evaluate the 3D error of the fine-tuned results. If the errors exceed a threshold, new control points should be introduced around the positions having an excessively large error. This validation flow continues repeatedly until all check points pass the verification.

### 4.4. Trajectory Refinement by NDT and Reconstruction of the Color Point Cloud into a Pcd File

SLAM is a popular technique in autonomous vehicle localization, which takes advantage of precise distance measurement from Lidar for matching with the features extracted from point cloud to estimate self-position. There are many methods to extract features for localization purpose, for example, the normal distribution transform (NDT), etc. More accurate features allow the MMS to estimate its own positions with high accuracy. Lidar-based localization methods can overcome the limitation of GNSS/IMU positioning systems as long as features in the point cloud are sufficient for Lidar localization. In the proposed system, the frequency of NDT localization is 15 Hz while the GNSS/IMU produces the location points at 200 Hz. In order to refine the trajectory by NDT localization, we set a threshold to determine the distance difference $D_{\left(x,y,z\right)}$ between the paired NDT location point $p_{NDT}$ and GNSS/IMU position $p_{TM2}$ by matched timestamp. When the absolute value of $D_{\left(x,y,z\right)}$ is greater than the threshold, the current and successive points of the GNSS/IMU location points $p_{TM2}$ are adjusted until next NDT localization is performed. The refined trajectory $P_{RFTM2}$ can be formed as:

(9) $$P_{RFTM2}(t_{NDT}+t_{offset})=P_{TM2}(t_{NDT}+t_{offset})+D_{\left(x,y,z\right)}(t_{NDT}),\;\mathrm{if}\;\left\|D_{\left(x,y,z\right)}(t_{NDT})\right\|>threshold,$$

where $P_{RFTM2}$: the refined trajectory; $P_{TM2}$: GNSS/IMU location points at 200 Hz, $D_{\left(x,y,z\right)}$: distance difference between the NDT location point $p_{NDT}$ and the GNSS/IMU position $p_{TM2}$ by matched timestamps; $t_{NDT}$: timestamp of NDT localization points and $t_{offset}$: time offset since the last $t_{NDT}$ has been obtained. In the proposed platform, $t_{offset}$ is $0\sim \frac{200}{15}$.

**Figure 15:**
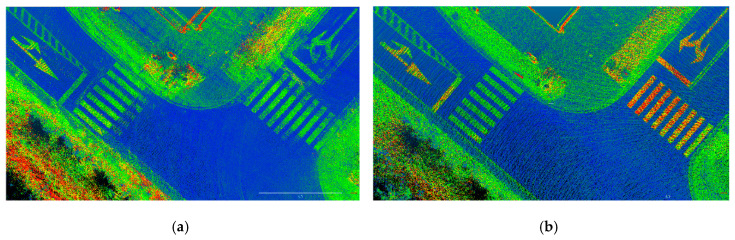
Top views of intensity point cloud showing (**a**) multi-scan dispersion effect due to GNSS/IMU trajectory error; (**b**) multi-scan dispersion issue is solved by NDT trajectory refinement.

In practice, multi-scans for areas like intersections or highways are inevitable, resulting in dispersion issues for point cloud reconstruction due to GNSS/IMU trajectory errors. According to Equation (9), we set the threshold to 10 cm to work out the refined trajectory $P_{RFTM2}$ and then reconstruct the point cloud. The dispersion issue of the point cloud can be properly addressed in the end. Figure 15 shows the improvement to solve the dispersion issue by the proposed NDT trajectory refinement method. In this section, we have described the methodology to accomplish 3 key tasks for color point cloud reconstruction:

(1) We correct the system time drift of the computing platform by a PPS signal to align timestamps for each sensor.
(2) We utilize the CalibrationToolkit with a calibration board to determine the extrinsic parameters between Lidar and camera sensors then compose the color into geo-coordinate point cloud.
(3) NDT localization is used to refine the trajectory based on an adjusted point cloud, solving the dispersion issue due to GNSS/IMU trajectory error to reconstruct the color point cloud.

## 5. Experimental Results

**Figure 16:**
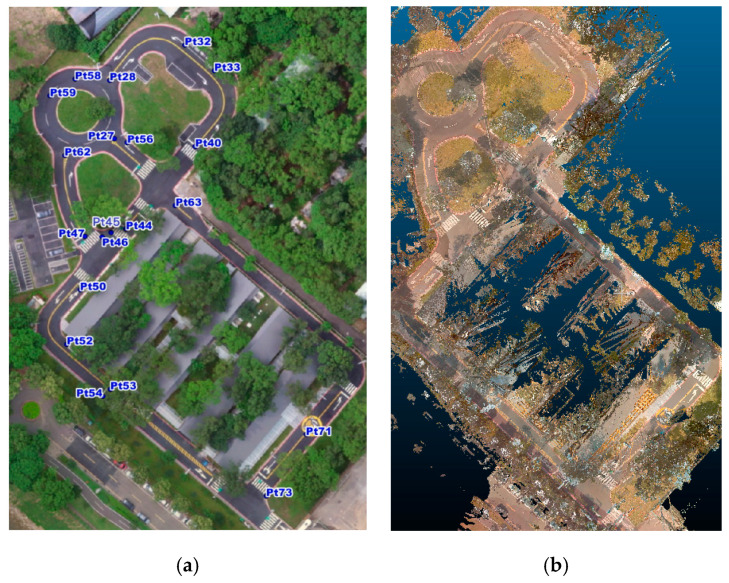
(**a**) Orthophoto with check points of the test area; (**b**) Top view of color point cloud reconstructed by the proposed method.

Experiments took place in the Hutoushan Innovation Hub [38] of Taiwan, which provides a closed area to carry out autonomous driving experiments. Figure 16a shows an orthophoto of the test area where the check points are indicated and Figure 16b shows a top view of the color point cloud reconstructed by the proposed method. We conduct extensive experiments to evaluate the thickness of Lidar points, position difference between color and intensity point clouds, and absolute position assessment of the color point cloud, respectively.

### 5.1. Point Cloud Thickness Assessment 

**Figure 17:**
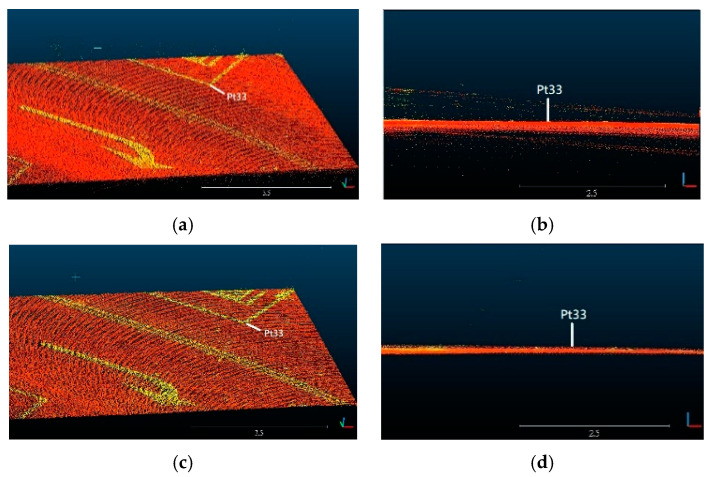
Top front view and side view of the Lidar points of an arrow sample near control point Pt33 by (**a**,**b**) conventional GNSS/IMU method [24] and (**c**,**d**) proposed point cloud reconstruction method.

**Figure 18:**
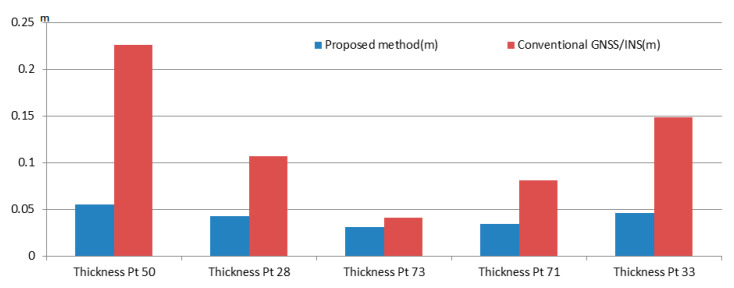
Thickness assessment of Lidar points for five sample arrows in Figure 16a.

To evaluate the thickness of Lidar points, we randomly pick five arrow samples on the ground from Figure 16a to measure the thickness of Lidar points. Figure 17 shows a top front view and side view of the Lidar points of an arrow sample near check point Pt33 in the point cloud by a conventional GNSS/IMU method [24] and the proposed point cloud reconstruction method. It can be seen that the thickness of the Lidar points obtained by the proposed method is thinner and more concentrated than that by the conventional method. Figure 18 illustrates the thickness of Lidar points of five sample arrows from Figure 16a, where the thickness of the Lidar points by the proposed point cloud reconstruction method has been significantly reduced into a few centimeters, which is desired for extracting precise height information.

### 5.2. Position Difference between Color and Intensity Point Clouds

**Figure 19:**
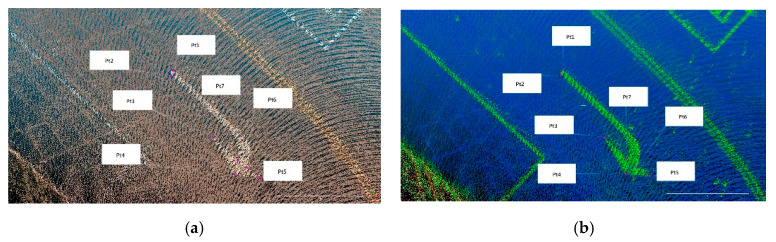
Top views of anchor points in (**a**) color point cloud; (**b**) intensity point cloud.

**Figure 20:**
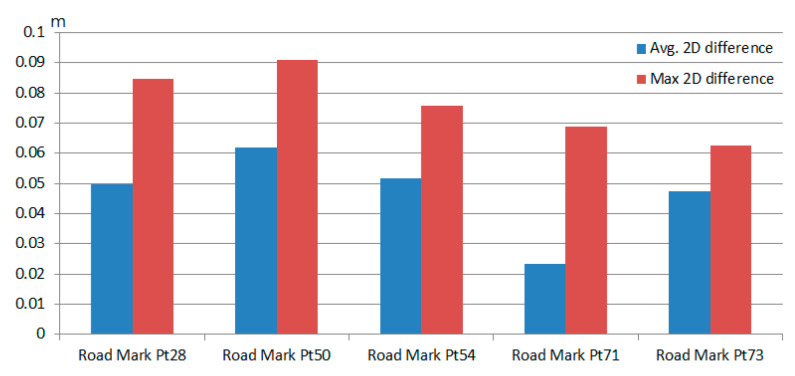
2D position difference of five road marks on the road surface in color and intensity point clouds.

Figure 19 shows seven anchor points of a sample road mark, which are manually marked in both color point cloud and intensity point cloud at the same locations for 2D position difference calculation. A total of five road marks around control points Pt28, Pt50, Pt54, Pt71 and Pt73 in Figure 16a are chosen for assessment. Figure 20 shows the average 2D position difference of marked anchor points between color and intensity point clouds of each road mark. The average position difference among the five chosen road marks is 4.6 cm. From Figure 20, it can be seen that road mark around Pt 50 has a larger position difference of 9.1 cm. This larger position difference is observed most likely when MMS is moving under a certain speed condition. Or, the capture time of the image frame and firing time of Lidar point reach their upper/lower bound of ±15 ms. Furthermore, a minor error due to manually marking the anchor points or calibration might also contribute to the final results.

### 5.3. Absolute Position Assessment

**Figure 21:**
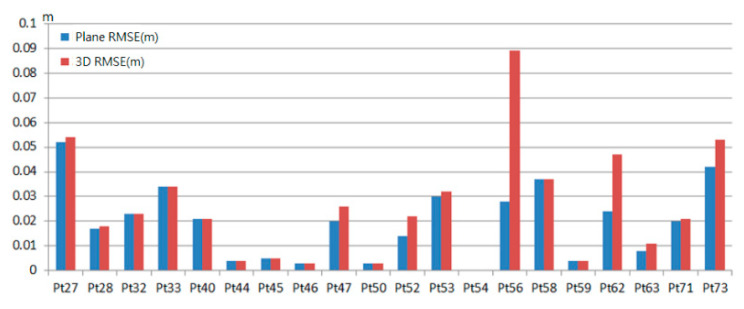
Plane and 3D RMSE of the check points.

For absolute position assessment, we measured 20 check points spreading in the test area shown in Figure 16a. The coordinate format of control points and color point cloud are unified into TM2 coordinate format based on TWD97 system for absolute position assessment. Figure 21 shows the accuracy of the proposed method in reconstructing the color point cloud, where average plane and 3D RMSE errors are 0.019 m and 0.025 m, respectively. According to the assessment results in the test area, we conclude that absolute RMSE can be controlled within 10 cm by the proposed method.

In this section, we have presented the reconstruction of color point cloud according to the methods we proposed. Through the thickness assessment, we’ve shown the proposed method benefits the reconstruction of point cloud in comparison to conventional GNSS/IMU approach. Position error of a few centimeters can be found when the RGB attributes are composed into the Lidar points. This error might result from the moving speed of the MMS, mismatch of image frame and Lidar point, manually marking the anchor points, or calibration.

**Figure 22:**
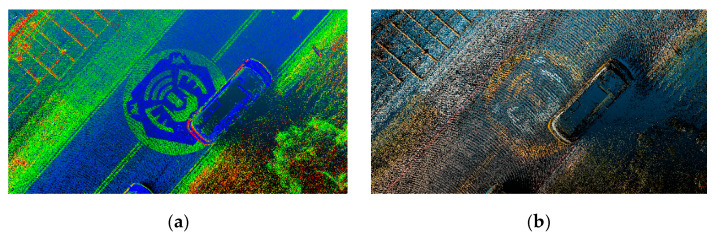
Top views showing the icon of the test area of the (**a**) intensity point cloud; (**b**) color point cloud constructed by the proposed method.

Figure 22 shows the top view of the color point cloud is clear and sufficient for people to identify the road surface attributes when drawing vector elements in HD map. According to the absolute error assessment, we can be sure that the color point cloud is reconstructed in high position accuracy.

**Figure 23:**
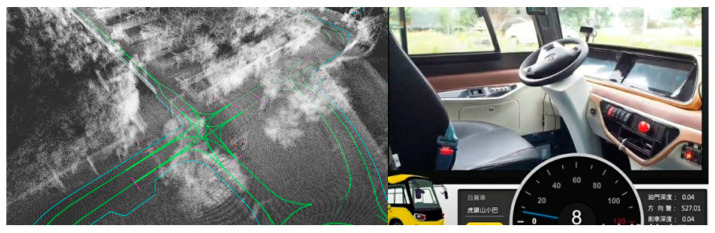
HD map established by color point cloud and vectors for use by an autonomous bus in the test area.

In summary, the MMS we proposed in this paper aims to reconstruct an accurate color point cloud to help create and manage vectors with coordinate information in HD map for autonomous driving. Table 1 shows a comparison between survey-grade MMS and the proposed MMS. It is easy to see the proposed MMS has low cost and high effectiveness benefits to penetrate the complicated processes by the proposed hardware integration and software implementation for color point cloud reconstruction. Figure 23 shows the color point cloud reconstructed by the proposed method was converted into a specific HD map format for use by an autonomous bus, which worked properly in the test area.

## 6. Conclusions

In this paper, we have presented a cost effective mobile mapping system to generate a color point cloud, in which an PX2 is employed as the computing platform for saving physical space and power consumption to perform multiple computation tasks in the vehicle, and a consumer-grade GMSL camera is chosen to gain a higher frame rate for color registration. By using the PPS signal, precise time synchronization can be obtained between the non-survey grade Lidar and consumer-grade GMSL camera. Through a calibration process between Lidar and camera, color attributes can be composed into the corresponding Lidar points in an accurate manner. To compensate the disadvantage of GNSS/IMU, we established control points to make adjustment to the point cloud constructed. Taking advantage of Lidar SLAM localization method, we can produce a refined trajectory based on which more accurate color point cloud can be reconstructed in the end. As demonstrated in the experiments, the thickness of the point cloud is much thinner than that by conventional methods using GNSS/IMU alone, which is desired for extracting precise height information required by HD map. Furthermore, we showed that the refined trajectory by NDT helps addressing the multi-scan dispersion issue to secure high-level absolute accuracy. Although there exist minor position errors in the colored point cloud, it has been confirmed that the proposed method can reconstruct color point cloud in centimeter-level accuracy, meeting the requirement of HD map for autonomous driving usage. In the future, we will investigate a challenging topic to automatically extract road/lane vectors and semantic information from the color point cloud to produce a HD map.

## Figures and Tables

**Table 1:** Comparison between survey-grade MMS and the proposed MMS.

Item	Survey-Grade MMS	Proposed MMS
Cost	High	Low (≅80,000 USD without SUV)
Post-processing	Normally operator has to tune the result in sub-steps	Can be done by one click after operator manually marking control points.
Point cloud density	High	Acceptable (≅4500 pts/$m^{2}$) and can be enhanced by adding more Lidars
Colored point cloud density	High	Acceptable (≅1400 pts/$m^{2}$) and can be enhanced by adding more cameras
Control points	Nice to have	Necessary (every 30 m~50 m)
